# Poisonings with Suicidal Intent Aged 0–21 Years Reported to Poison Centers 2003–12

**DOI:** 10.5811/westjem.2015.5.25459

**Published:** 2015-07-14

**Authors:** Sophia Sheikh, Phyllis Hendry, Sean Lynch, Colleen J. Kalynych, Petra Aldridge, Dale Kraemer

**Affiliations:** * University of Florida College of Medicine-Jacksonville, Department of Emergency Medicine, Jacksonville, Florida; † U.S. Department of Health and Human Services, Substance Abuse and Mental Health Services Administration Center, Rockville, Maryland; ‡ University of Florida College of Medicine-Jacksonville, Center for Health Equity and Quality Research, Jacksonville, Florida; § University of Florida College of Medicine-Jacksonville, Department of Neurology, Jacksonville, Florida

## Abstract

**Introduction:**

Few studies explore the clinical features of youth suicide by poisoning. The use of both social and clinical features of self-poisoning with suicidal intent could be helpful in enhancing existing and creating new prevention strategies. We sought to characterize self-poisonings with suicide intent in ages 0 to 21 years reported to three regional poison control centers from 2003–2012.

**Methods:**

This study was a blinded retrospective review of intentional self-poisonings by those age 21 or younger captured by the Poison Information Control Network. Age, sex, substance(s) used, medical outcome, management site, clinical effects, and therapies were described using counts and percentages and analyzed using chi-square tests. We analyzed the medical outcome ranging from no effect to death using the Wilcoxon rank-sum test. Serious medical outcome was defined as death or major outcome.

**Results:**

We analyzed a total of 29,737 cases. The majority were females (20,945;70.5%), of whom 274 (1.3%) were pregnant. Most cases were 15–18 year olds (15,520;52.2%). Many experienced no effects (9,068;30.5%) or minor medical outcomes (8,612;29%). Males had more serious medical outcomes (p<0.0001), but females were more likely to be admitted to a critical care unit (p<0.0001). There were 17 deaths (0.06%), most in males (10;p=0.008). Of the 52 substances reported in the death cases, 12 (23.1%) were analgesics. In eight (47.1%) of the deaths, over two substances were used. Overall, drowsiness/lethargy (7,097;19.3%) and single-dose charcoal (8,815;16.3%) were frequently reported. Nearly 20% were admitted to critical care units (5,727;19.3%) and 28.7% went to psychiatric facilities (8,523). Of those admitted to hospitals (8,203), nearly 70% (5,727) required critical care units. Almost half <10 years old were evaluated and released (43;47.2%). Of the 114 reported substances for this population, 22.8% involved psychotropic medications, 15.8% analgesics, and 14% Attention Deficit-Hyperactive Disorder (ADHD) medications. Analgesics (13,539;33.6%) were the most common medication category used by all age groups. Typically only one substance (20,549;69.1%) was used.

**Conclusion:**

Undiagnosed ADHD may be a potential underlying cause for self-harming behaviors in the very young. Gender-specific suicide prevention strategies may be more effective at identifying those at risk than traditional measures alone. Further study into admitting practices by emergency physicians is needed to understand the difference in critical care admission rates based on gender. Once identified to be at-risk for suicidal behavior, access to analgesics and psychotropics should be monitored by care-givers especially in those between the ages of 15–18.

## INTRODUCTION

Self-poisoning is a top cause of pediatric injury.^1^,^2^ The 1997–2002 National Hospital Ambulatory Medical Care Survey found the annual emergency department (ED) rate for self-harm in those aged 7–24 was 225.3 per 100,000.^3^ It has been reported across all age groups that for nearly every suicide there are 12–15 self-harm related ED visits.^4^ The mean charge for each ED visit related to self-harm was found to be $1,874 ($12,801 for visits resulting in hospital admission) with total U.S. hospitalization charges estimated to be $227.85 million.^2^ Early identification and effective prevention strategies may ease the health and financial burden for this preventable situation.

Studies have attempted to identify predictors for pediatric suicide. These include demographic and social factors, such as female gender, history of adoption, gay and lesbian youth, depression, history of previous suicide attempt, drug abuse, and poor social support.^1^,^5^,^6^ However, few studies have characterized the clinical features of youth suicide. The National Electronic Injury Surveillance System All Injury Program found that among the 1,197 nonfatal self-harm injury cases between ages 10–14 years treated in an ED from 2001–2003, nearly half of the cases involved either an over-the-counter (OTC) (28.3%) or prescription drug (21%).^7^ This study did not detail the OTC and prescription medication(s) used. A non-United States study identified analgesics as a frequent cause of pediatric poisoning from both accidental and intentional exposures, including misuse, abuse, and suicide.^8^

Most of the pediatric suicide literature focuses on demographic and social characteristics. Few studies explore the clinical features of youth suicide by poisoning. The use of both social and clinical features of self-poisoning with suicidal intent could be helpful in enhancing existing and creating new prevention strategies.

## METHODS

Calls made to the poison control centers (PCC) are received by certified poison specialists who are trained and prompted by the electronic charting system to ask for standardized data points. Data from the initial call and subsequent follow-up calls are recorded in a standardized electronic chart. Whether an exposure is a suspected suicide attempt is reported by the caller and is not a determination made by the specialist taking the call. The Florida Poison Information Control Network (FPICN) database houses all the electronic charts generated from every call made by the public and medical caregivers to each of the three Florida PCCs. This database can be queried to select charts of interest based on standard data points, such as age, gender, route of exposure to toxin, medical severity/outcome, clinical effects, treatments, type of toxin, management site, etc. These data points are collected for every call made to the poison center when possible and are consistent with the standardized coding system set forth by the American Association of PCCs.

We obtained institutional review board approval prior to initiation of the study. A blinded retrospective review of data collected by all three PCCs was performed. We collected data via a blinded query of the FPICN database. All human exposure cases coded as “intentional-suspected suicide” in those up to 21 years of age reported to a PCC from 2003 to 2012 were exported to Microsoft (MS) Excel^®^ 2010. Exclusion criteria included the following: (1) information calls (i.e. pill identification); (2) calls with an active clinical course; (3) reported exposures determined to not be clinically related or was a confirmed non-exposure by the PCC; (4) unintentional, intentional misuse, or intentional abuse exposures; and (5) suicide other than self-poisoning.

We collected and analyzed data on age, gender, medical outcome, management site, clinical effects, and therapies using MS Excel^®^ 2010 and SAS^®^ 9.3. Age, sex, substance(s) used, medical outcome, management site, clinical effects, and therapies were described using counts and percentages and analyzed using chi-square tests. We analyzed medical outcome using the Wilcoxon rank-sum test. The Wilcoxon rank-sum test effect size was 0.07. Gender data was missing for 26 cases (0.087% of all cases).

Serious medical outcome was defined as death or major outcome (Table 1). Because we collected clinical effects, treatment, and substance data as aggregate data for ages 10–21 years, we were thus unable to further analyze the data by gender.

## RESULTS

We analyzed a total of 29,737 cases. Gender data was missing for 26 cases. Table 1 describes demographic and health data. Table 2 lists the health data by gender. Of the 40,297 substances reported, analgesics (13,539;33.6%), psychotropic medications (10,710;26.6%), and antihistamines/cough and cold preparations (4,339;10.8%) were the top medication categories used. Youths typically used one substance (20,549;69.1%). Over three substances were used by 4.3% of the youths.

### Deaths

There were 17 deaths (0.06%), all over age 13, with more males (10;59%) resulting in death than females (p=0.008). Analgesics (12;23.1%) were most commonly reported. More than two substances were used in 8 (47.1%) deaths.

### Less than 10 years of age (91,<1%)

There was no significant difference in gender for this age group. Patients typically had minor clinical effects (31.9%). Drowsiness/lethargy was the top reported clinical effect (24.2%). Of the 114 reported substances for this population, 22.8% involved psychotropic medications, 15.8% analgesics, and 14% attention deficit-hyperactive disorder (ADHD) medications. Of the 118 different treatments given, single dose activate charcoal was most common (17.5%). Observation only was performed in 17.5% of the cases. Almost half were evaluated/released (43;47.2%), with 12% (11) requiring critical care unit admission, and 11% (10) to a psychiatric facility.

## DISCUSSION

Consistent with existing literature, we found that patients between the ages of 15–18 years to be particularly vulnerable to committing self-harm compared to other age groups.^3^ We also found a surprisingly high number of very young patients (less than age 10) attempting suicide. Unlike previous reported data, we did not find a difference in gender for this age group.^9^ Social stressors, psychiatric/behavioral disorders, and modeling adult behavior have been reported as potential causes for self-harm in the very young.^9^ Patients in this age group typically are not considered to have the means nor ability to carry out pre-planned self-harming behaviors as they usually receive more adult supervision than older children and are cognitively less able to devise and implement a plan with the intent of self- injury. It is speculated that these acts are the result of impulsivity. Impulsivity is a defining feature of ADHD. We found ADHD medications to be frequently used by our younger population (less than 10 years old) for committing self-harm. Impulsivity may be the primary contributing factor in these cases as depression is rare in this age group. Interestingly, children with ADHD (especially females) between the ages of 4–6 years have been shown to be at increased risk for developing depression and suicidality through age 18.^10^ Thus, with increasing age impulsivity may take a less influencing role compared to depression for committing self-harming behavior. ADHD, along with other mental health conditions, may predispose patients to self-harm now and in the future. Therefore, we suggest that screening for ADHD, in addition to the other screening measures, should be considered in the very young when presenting with self-harming behavior. Further study into this potential cause of self-harm in this population is needed.

Our study is consistent with others that have suggested gender differences in suicide-related behaviors. Some reported differences are that males tend to be unmarried, unemployed, consume alcohol, marijuana, or tobacco.^11^ We found males developed more serious medical outcomes including death, but committed self-poisonings less often. This conflicts with previous findings showing a greater proportion of male ED visits for self-inflicted injury were discharged compared to females. This implies less morbidity in males. However, this study also reported that more males died in the ED than females.^2^ Given the presence of gender differences when it comes to suicide-related behaviors and outcomes, it has been proposed that the use of traditional suicide ideation/depression screening measures alone may not be adequate. Females tend to score higher on traditional suicidal ideation/depression measures, whereas alcoholism/substance abuse (risk-taking behaviors) are more commonly diagnosed in males.^11^–^12^ Instead a combination of traditional and risk-taking/life-diminishing screening should be performed to increase identification of those at-risk for both genders.^12^

Most of our patients did not develop significant symptoms and required simple therapies. Despite this, most of the 8,203 (27.6% of total patients) admissions were sent to a critical care setting (5727,69.8%). Conversely, a study looking at mostly adult patients (1,573;76.4%) found that only 31% of the one-third admitted as a result of self-harm went to a critical care unit.^3^ Interestingly, we found that females were more likely to be admitted to a critical care unit even though males tended to have more serious medical outcomes. Reasons for this discrepancy in admitting practices for pediatric compared to adult self-harm patients and for pediatric female compared to pediatric males are unclear from our data. This difference may be due to physicians opting for more conservative management for pediatric suicide cases compared to similar adult cases. However this does not explain differences in admitting practices for pediatric patients based on gender. Often the decision for admission to a critical care setting is made by the treating emergency physician. Further study into the management and disposition decisions made by treating emergency physicians may help shed light on these practices.

Most patients used only one medication when attempting self-harm. However, in cases resulting in death, nearly half used more than one substance. For death and non-death resulting cases, analgesics were most commonly used for committing self-harm. Self-poisoning by non-prescription analgesics may be a popular choice by the pediatric population because of accessibility. Consistent with existing literature, we found that psychotropic medications were also popular choices.^2^ The presence of a mental health disorder has been shown to confer a significant risk for committing self-harm. It is possible that certain suicidal patients, such as those on antidepressants, may be more likely to use their own prescribed medications to commit self-harm. However, further study would need to be done to explore this possibility. Parents of these at-risk populations should be educated regarding limiting access to OTC and prescription medications.

## CONCLUSION

Undiagnosed ADHD may be a potential underlying cause for self-harming behaviors in the very young. Gender-specific suicide prevention strategies may be more effective at identifying those at risk than traditional measures alone. Further study into admitting practices by emergency physicians is needed to understand the difference in critical care admission rates based on gender. Once identified to be at-risk for suicidal behavior, access to analgesics and psychotropics should be monitored by care-givers especially in those between ages 15–18.

## LIMITATIONS

The limitations of this study are inherent to PCC data such as second-hand passive reporting, convenience sampling, and incomplete data as all information for a case may not have been reported and not call cases are reported to PCCs. Additionally, as this was a retrospective study bias and potential confounders may not have been fully accounted for. Also, the study population may not accurately reflect the entire US and thus our conclusions may not be generalizable outside the study region. Since calls made to PCCs are voluntary all potential cases may not have been captured. It is also possible that treating medical staff may be more likely to call in cases of serious overdose. However, despite this we still found that nearly 60% of reported cases had either minor medical outcome or no symptoms.

## Figures and Tables

**Table 1:** Demographic and health data of self-poisonings with suicidal intent in young patients (N=29,737).

Demographics	N (%)
Females	20,945 (70.5%)
Pregnant	274 (1.3%)
Age (years)	
<10	91 (0.31%)
10–14	4,454 (15%)
15–18	15,520 (52.2%)
19–21	9,672 (32.5%)
Health data	
Medical outcome^1^,^2^	
No effect-The patient did not develop any sign or symptoms as a result of the exposure	9,068 (30.5%)
Minor-The patient developed minimally bothersome symptoms (e.g., self-limited gastrointestinal symptoms, drowsiness, skin irritation, sinus tachycardia without hypotension) that resolved with no residual effects.	8,612 (29%)
Moderate-The patient exhibited more pronounced, more prolonged, or more systemic symptoms than minor but not life-threatening and no residual symptoms (e.g., corneal abrasion, acid-base disturbance, high fever, disorientation, responsive hypotension, brief seizure). Usually treatment was required.	7,136 (24%)
Major-The patient exhibited symptoms that were life-threatening or resulted in residual disability (e.g., repeated seizure, respiratory compromise requiring intubation, ventricular tachycardia with hypotension, cardiac/respiratory arrest, esophageal stricture).	719 (2.4%)
Death-The patient died as a result of the exposure or as a direct complication of the exposure.	17 (0.06%)
Unable to follow-potentially toxic exposure	4,185 (14.1%)
Management site	
Treated/evaluated and released	8,507 (28.6%)
Managed on site (non-healthcare facility)	31 (0.1%)
Admission to hospital	8,203 (27.6%)
Non-critical care unit	2,476 (30.2%)
Critical care unit	5,727 (69.8%)
Admission to psychiatric facility	8,523 (28.7%)
Patient lost to follow up/left against medical advice	1,767 (5.9%)
Other/unknown	2,706 (9.1%)
Clinical effects**	36,776
Drowsiness/lethargy	7,097 (19.3%)
Tachycardia	5,789 (15.7%)
Vomiting	3,790 (10.3%)
Treatment**	
Single-dose activated charcoal	8,815 (16.3%)
Intravenous fluids	7,786 (14.4%)

1 Medical outcome categories are consistent with those of the American Association of Poison Control Centers.

2 Serious outcome defined as death or major effect.

* Gender data missing for 26 cases.

** Collected as aggregate data for ages over 10 years.

**Table 2:** Health data analysis by gender.

	Male N(%)	Female N(%)	Overall N(%)	p-value
Death				
No	8,756 (99.9)	20,938 (99.9)	29,694 (99.9)	0.008
Yes	10 (0.1)	7 (0.03)	17 (0.1)	
Management site				
Admitted to critical care unit	1,922 (21.9)	3,804 (18.2)	5,726 (19.3)	<0.0001
Admitted to noncritical care unit	730 (8.3)	1,745 (8.3)	2,475 (8.3)	
Admitted to psychiatric facility	2,304 (26.3)	6,218 (29.7)	8,522 (28.7)	
Managed on site (non-healthcare facility)	13 (0.15)	18 (0.1)	31 (0.1)	
Other/unknown/refused	838 (9.6)	1,858 (8.9)	2,696 (9.1)	
Patient lost to follow up/left against medical advice	541 (6.2)	1,216 (5.8)	1,757 (5.9)	
Treated/evaluated and released	2,418 (27.6)	6,086 (29.1)	8,504 (28.6)	
Medical outcome^1^				
Death	10 (0.1)	7 (<0.0)	17 (0.1)	<0.0001
Major effect	280 (3.2)	439 (2.1)	719 (2.4)	
Minor effect	2,417 (27.6)	6,192 (29.6)	8,609 (29)	
Moderate effect	2,452 (28)	4,683 (22.4)	7,135 (24)	
No effect-nontoxic exposure	2,312 (26.4)	6,753 (32.2)	9,065 (30.5)	
Unable to follow-potentially toxic exposure	1,295 (14.8)	2,871 (13.7)	4,166 (14)	
Serious outcome^2^				
No	8,476 (96.7)	20,499 (97.9)	28,975 (97.5)	<0.0001
Yes	290 (3.3)	446 (2.1)	736 (2.5)	

All tests performed using chi-squared analysis.

1 Medical outcome categories are consistent with those of the American Association of Poison Control Centers.

2 Serious outcome defined as death or major effect.
